# The IMPDH cytoophidium couples metabolism and fetal development in mice

**DOI:** 10.1007/s00018-024-05233-z

**Published:** 2024-05-08

**Authors:** Min Peng, Gerson D. Keppeke, Li-Kuang Tsai, Chia-Chun Chang, Ji-Long Liu, Li-Ying Sung

**Affiliations:** 1 Institute of Biotechnology, National Taiwan University, Taipei, 106 Taiwan; 2 School of Life Science and Technology, ShanghaiTech University, Shanghai, 201210 China; 3 Departamento de Ciencias Biomédicas, Facultad de Medicina, Universidad Católica del Norte, Coquimbo, Chile; 4 Department of Physiology, Anatomy and Genetics, University of Oxford, Oxford, OX1 3PT UK; 5 Center for Developmental Biology and Regenerative Medicine, National Taiwan University, Taipei, 106 Taiwan; 6 Center for Biotechnology, National Taiwan University, Taipei, 106 Taiwan; 7 Agricultural Biotechnology Research Center, Academia Sinica, Taipei, 115 Taiwan

**Keywords:** IMPDH, Cytoophidium, Enzyme polymerisation, GTP biosynthesis, Pluripotent stem cells, Embryonic development

## Abstract

The cytoophidium is an evolutionarily conserved subcellular structure formed by filamentous polymers of metabolic enzymes. In vertebrates, inosine monophosphate dehydrogenase (IMPDH), which catalyses the rate-limiting step in guanosine triphosphate (GTP) biosynthesis, is one of the best-known cytoophidium-forming enzymes. Formation of the cytoophidium has been proposed to alleviate the inhibition of IMPDH, thereby facilitating GTP production to support the rapid proliferation of certain cell types such as lymphocytes, cancer cells and pluripotent stem cells (PSCs). However, past studies lacked appropriate models to elucidate the significance of IMPDH cytoophidium under normal physiological conditions. In this study, we demonstrate that the presence of IMPDH cytoophidium in mouse PSCs correlates with their metabolic status rather than pluripotency. By introducing IMPDH2 Y12C point mutation through genome editing, we established mouse embryonic stem cell (ESC) lines incapable of forming IMPDH polymers and the cytoophidium. Our data indicate an important role of IMPDH cytoophidium in sustaining a positive feedback loop that couples nucleotide biosynthesis with upstream metabolic pathways. Additionally, we find that IMPDH2 Y12C mutation leads to decreased cell proliferation and increased DNA damage in teratomas, as well as impaired embryo development following blastocoel injection. Further analysis shows that IMPDH cytoophidium assembly in mouse embryonic development begins after implantation and gradually increases throughout fetal development. These findings provide insights into the regulation of IMPDH polymerisation in embryogenesis and its significance in coordinating cell metabolism and development.

**Supplementary Information:**

The online version contains supplementary material available at 10.1007/s00018-024-05233-z.

## Introduction

Nucleotides participate in a wide variety of biochemical processes in cells. They serve not only as the building blocks of DNA and RNA but also as energy sources in signalling pathways and essential cofactors in metabolic reactions. Maintaining a delicate balance of nucleotide pools is vital for normal cell physiology, as disruptions can lead to cellular abnormalities, highlighting the fundamental importance of nucleotide biosynthesis.

Inosine monophosphate dehydrogenase (IMPDH) catalyses the conversion of inosine monophosphate (IMP) to xanthosine monophosphate (XMP), a committed and rate-limiting step in guanine nucleotide biosynthesis. There are two isoforms, IMPDH1 and IMPDH2, sharing 84% sequence identity in human genome and each playing distinct physiological roles [1]. While the loss of IMPDH1 leads to retinopathy, the absence of IMPDH2 results in embryonic lethality [2, 3]. Moreover, IMPDH1 maintains a constantly low level, whereas IMPDH2 is widely present in various tissues and upregulated in rapidly dividing cells [4, 5].

IMPDH has been revealed to be regulated by polymerisation and the polymers can self-assemble into a filamentous structure, termed the cytoophidium (plural “cytoophidia”, meaning cellular snakes in Greek) in vertebrate models [6–9]. The activity of IMPDH is inhibited by GTP binding; however, polymer formation can alleviate this inhibition, thereby maintaining enzyme activity under conditions of elevated GTP levels. Disruption of the IMPDH polymerisation interface with the Y12A point mutation suppresses catalytic activity, particularly when GTP pools expansion is needed [7, 10].

In the cell, cytoophidium assembly is controlled by the amount of IMPDH polymers and molecular crowding [9]. The presence of cytoophidium reflects the upregulation of GTP production and may also protect IMPDH from degradation [6, 9]. Therefore, the assembly of IMPDH cytoophidium has been suggested to play important roles in sustaining GTP levels and promoting cell proliferation [6, 7, 11].

IMPDH cytoophidia are naturally present in various animal tissues and correlate with the active metabolic state of cells [6, 8, 12–15]. In mouse embryonic stem cells (ESCs) and induced pluripotent stem cells (iPSCs), IMPDH cytoophidia are prevalent under normal culture conditions but disassemble upon differentiation, implying their association with the metabolic signatures of pluripotent stem cells (PSCs) [11, 16].

While most differentiated cells predominantly rely on mitochondrial oxidative phosphorylation for energy production, PSCs prefer aerobic glycolysis like some cancer cells [17, 18]. This metabolic adaptation enables proliferative cells to efficiently generate ATP and synthesise biomass, including amino acids, lipids and nucleotides, supporting their heightened anabolic demands during proliferation [19, 20]. Considering the intricate link between nucleotide precursor biosynthesis, glycolysis and pentose phosphate pathway (PPP), we suspect that IMPDH polymerisation and cytoophidium assembly may participate in coordinating nucleotide synthesis with upstream metabolic pathways.

Here we have explored the relationship between IMPDH cytoophidium formation and metabolic regulation in mouse PSCs. Inhibition of glycolysis or the PPP, or induction of PSC differentiation, led to IMPDH cytoophidium disassembly. Conversely, during iPSC reprogramming, cytoophidium assembly occurred alongside the upregulation of key genes in glycolysis and PPP but not pluripotency genes. Mutant ESCs with an IMPDH2 Y12C point mutation displayed a no-cytoophidium phenotype, accompanied by reduced expression of *c-Myc* and genes related to glycolysis, PPP and purine nucleotide synthesis. The addition of guanosine reversed this downregulation. Furthermore, mutant ESCs formed smaller teratomas with decreased proliferation and increased DNA damage post-transplantation, resembling the metabolic shift observed in cultured mutant ESCs. Blastocoel injection revealed significantly fewer live births and no chimeras from mutant ESCs, indicating compromised embryonic development contribution. The presence of IMPDH cytoophidium in most tissues of normal mouse fetuses suggests its significance in coordinating cell metabolism and indispensable roles in mammalian embryogenesis and fetal development.

## Materials and methods

### Cell culture

Mouse PSCs were cultured on Mitomycin C (M4287, Sigma-Aldrich)-inactivated mouse embryonic fibroblast (MEF) feeders in ESC medium consisting of Dulbecco’s modified Eagle’s medium with high glucose, pyruvate (DMEM, 11995065, Gibco), 15% fetal bovine serum (FBS, TMS-013-BKR, Millipore), 1,000 U/mL mLIF (ESG1107, Millipore), 1% Penicillin-Streptomycin (15140-122, Gibco), 2 mM L-glutamine (A29168-01, Gibco), MEM NEAA (11140-050, Gibco), 1 mM sodium pyruvate (11360-070, Gibco) and 0.1 mM 2-mercaptoethanol (ES-007-E, Millipore). The cells were maintained in a 37 °C humid incubator with 5% CO_2_, and the culture medium was changed every two days. Retinoic acid (RA, R2625, Sigma-Aldrich), 2-deoxyglucose (2DG, D6134, Sigma-Aldrich), 6-aminonicotinamide (6AN, A68203, Sigma-Aldrich) and guanosine (G6264, Sigma-Aldrich) were used in the experiments.

### iPSC generation

iPSCs were generated from MEFs by retroviral infection. To produce retroviruses, Platinum-E (Plat-E) cells were cultured in MEF medium containing DMEM supplemented with 10% FBS. The pMX-based *Oct4/Pou5f1*, *Sox2*, *Klf4* and *c-Myc* plasmids, purchased from Addgene, were transfected into Plat-E cells at 80% cell confluence using TurboFect Transfection Reagent (Thermo Scientific) following the manufacturer’s instructions. In brief, 2 µg of DNA and 6 µL of transfection reagent were mixed in 400 µL of serum-free DMEM. After a 15–20 min incubation, the transfection reagent/DNA mixture was added drop-wise to each well of a 6-well plate (353046, Falcon). The virus-containing supernatants were collected 48 h post-transfection and filtered through 0.45 μm cellulose acetate membrane filters (Sigma-Aldrich). These retroviruses encoding POU5F1/OCT4, SOX2, KLF4 and c-Myc were combined at equivalent ratios with 8 µg/mL polybrene (H9268, Sigma-Aldrich) before infecting MEFs at Day (D) 0. MEFs were seeded at a density of 1 × 10^5 cells per well of a six-well plate and maintained in MEF medium one day before infection. After 2 days of retroviral infection, the culture medium was replaced with MEF medium for 3 days and then changed to ESC medium on D5. The cells were subsequently replated onto MEF feeders at a density of 2 × 10^5 cells per six-well dish on D7. After four weeks post-infection, colonies were manually picked using sterile pipet tips for iPSC line derivation.

### Establishment of mutant ESCs by ABEmax base editing

pCMV_ABEmax-T2A-mCherry [21] and pGL3-U6-sgRNA-PGK-Puro (#51,133, Addgene) plasmids were used for generating mutant ESC lines. Oligos for sgRNA expression plasmid (see Supplementary Table 1) were annealed and cloned into the BsaI sites of pGL3-U6-sgRNA-PGK-Puro. These plasmids were then transfected into ESCs when they reached 60% confluence using Lipofectamine Stem Transfection Reagent (Invitrogen). Briefly, 1 µL of transfection reagent was mixed with 25 µL of serum-free DMEM, and 500 ng of DNA was mixed with 25 µL of serum-free DMEM separately. The two solutions were then combined and incubated for 10 min before being added to each well of a 24-well plate (353047, Falcon). Two days after transfection, ESCs expressing mCherry were collected as a pool by BD FACS (Fluorescence-Activated Cell Sorter) Aria III Sorter. After subculture, individual colonies derived from single cells were manually picked by sterile pipet tips for sequencing of the targeted DNA region to identify mutant ESC lines.

### Animals

All procedures related to the maintenance, care and use of animals were approved by the Institutional Animal Care and Use Committee of National Taiwan University, under protocol number NTU-107-EL-216. Crl: CD1(ICR) mice obtained from BioLASCO Taiwan Co., Ltd. were utilised in this study.

### Teratoma formation

Following treatment with a 0.05% trypsin-EDTA solution (25200056, Gibco), a total of 6 × 10^5 cells were concentrated in 50 µl of culture medium and injected into the thigh muscles of 10-week-old male nude mice using a 26 G X ½” hypodermic needle. The harvested transplants, obtained five weeks post-transplantation, underwent immediate freezing in liquid nitrogen. A portion of the tissues was promptly embedded in O.C.T. compound (4583, Tissue-Tek) upon collection. Subsequently, all tissues were stored at -80 °C before use.

### Whole-mounted mouse embryos and embryo cryosections preparation

Embryos were obtained through mating superovulated ICR females with males, confirmed by the presence of the vaginal plug. Fertilised 1-cell zygotes were harvested 18 h post-human chorionic gonadotropin (hCG), dissected from the oviduct’s ampulla. The cumulus cells of the embryo complexes were dispersed using 300 mg/mL of hyaluronidase. Embryos ranging from two-cell to blastocyst stages were collected through oviduct and uterine horn flushing. Preimplantation embryos were fixed in 4% PFA for 20 min at 25 °C. Embryos at embryonic day (E) 7.5–9.5 were isolated from the uterine horn and fixed in 4% paraformaldehyde (PFA) overnight at 4 °C, while those older than E10.5 were immediately embedded in O.C.T. compound (4583, Tissue-Tek) and stored at -80 °C before cryosectioning.

### Cryosection

Teratoma tissue samples and embryos older than E10.5 were subjected to cryosectioning using the Leica CM1950 cryostat. The resulting sections were transferred and mounted onto glass slides, which had been pre-treated with silane (5116, MUTO PURE CHEMICALS).

### EdU labelling

Cultured cells were seeded onto coverslips and exposed to 10 µM of EdU for 10 min to label newly synthesised DNA when they reached 60% confluence. Following EdU incubation, cells were fixed with 4% PFA, washed twice with a wash solution containing 3% bovine serum albumin (BSA, A9647, Sigma-Aldrich) in PBS, and then incubated with permeabilisation buffer consisting of 0.5% Triton X-100 in PBS for 20 min. Subsequently, an azide-based Click-iT reaction was performed according to the manufacturer’s protocol (Invitrogen) to conjugate the Alexa Fluor 647 molecule to the EdU. Briefly, after washing the cells twice with the wash solution, they were incubated with a Click-iT reaction cocktail composed of 430 µL of 1X Click-iT EdU reaction buffer, 20 µL of CuSO_4_, 1.2 µL of Alexa Fluor azide and 50 µL of 1X Click-iT EdU buffer additive. After a 30-min incubation in the dark, cells were washed with the wash solution and ready for antibody labelling and nucleus staining, following the same procedure as the immunofluorescent staining.

### Immunofluorescent staining

The fixed samples underwent a 2-hour incubation at room temperature with the primary antibody in a solution composed of 2.5% BSA and 0.25% Triton-X100 in PBS. Subsequently, PBS washes were performed, followed by a minimum 2-hour incubation with the secondary antibody. After the secondary antibody reaction, nucleic acid was labelled through staining with DAPI (D9542, Sigma-Aldrich). Subsequent PBS washes were carried out, and the samples were mounted in PBS for imaging. Tissue staining was conducted alongside control sections stained only with secondary antibodies. Antibodies employed in this study included mouse anti-IMPDH1 monoclonal antibody (ab55297, Abcam), rabbit anti-IMPDH2 polyclonal antibody (12948-1-AP, ProteinTech), rabbit anti-NANOG polyclonal antibody (REC-RCAB002P-F, Cosmobio), rabbit anti-phospho-Akt1 (S473) monoclonal antibody (AP0637, Abclonal), mouse anti-OCT3/4 monoclonal antibody (sc-5279, Santa Cruz Biotechnology), rabbit anti-phospho-histone H3 (S10) polyclonal antibody (p-H3, GTX128116, GeneTex), Alexa Fluor 488-conjugated goat anti-rabbit IgG polyclonal antibody (A11034, Invitrogen), Alexa Fluor 488-conjugated goat anti-mouse IgG polyclonal antibody (A11029, Invitrogen), Alexa Fluor 647-conjugated goat anti-rabbit IgG polyclonal antibody (A21244, Invitrogen) and Alexa 647-conjugated goat anti-mouse IgG polyclonal antibody (A21235, Invitrogen). All antibodies were applied at a 1:500 dilution. Images were captured using a Leica TCS SP5 II laser-scanning confocal microscope at room temperature, utilising Leica Application Suite Advanced Fluorescence (LAS AF) software. Excitation of DAPI was performed with a diode laser (405 nm), while Alexa Fluor 488 and Alexa Fluor 647 were excited using an argon-ion laser (488 nm) and a red helium-neon laser (647 nm), respectively. Imaging parameters included argon laser power set at 30%, XYZ acquisition mode, image format of 1024 × 1024 pixels, speed of 400 Hz and zoom factor of 1, using either 40x/1.25 or 63x/1.4 oil immersion objectives.

### Gene expression

RNA extraction was carried out using the GENEzol TriRNA Pure Kit (GZX100, Geneaid) in accordance with the provided instructions. The subsequent preparation of the cDNA solution involved the use of GoScript Reverse Transcriptase (A2801, Promega). In the context of real-time PCR analysis, KAPA SYBR® FAST qPCR Master Mix (KK4609, Roche) was employed, following the manufacturer’s protocol. Real-time qPCR was performed on a Bio-Rad CFX384 qPCR System, involving 40 cycles of 3 s at 95 °C and 20 s at 60 °C, along with a thermal denaturing step to generate dissociation curves for validating amplification specificity. The normalisation of gene expression utilised the CT value of *Actin*. Refer to Supplementary Table 1 for a comprehensive list of the primers.

### Western blotting

Cell lysates were acquired by treating cells with RIPA lysis buffer (20-188, Millipore), and the protein concentration was determined using a Bio-Rad Protein Assay Kit (5000002, Bio-Rad). These lysates were then loaded onto a 12% polyacrylamide gel and transferred onto a PVDF membrane (GE Healthcare). For immunolabelling, primary and secondary antibodies were diluted in PBST with 5% milk and left to incubate overnight. The antibody labelling was visualised using the SuperSignal West Pico PLUS Chemiluminescent Substrate (34579, Thermo Scientific) and captured with a chemiluminescence imaging system (GeneGnome XRQ, Syngene). The antibodies used included rabbit anti-IMPDH2 polyclonal antibody (1:10000, 12948-1-AP, ProteinTech), rabbit anti-phospho-Akt1 (S473) monoclonal antibody (1:3000, AP0637, Abclonal), rabbit anti-Akt (Pan) polyclonal antibody (1:3000, IR171-666, iREAL Biotechnology), mouse anti-OCT3/4 monoclonal antibody (1:3000, sc-5279, Santa Cruz Biotechnology), HRP-conjugated mouse anti-β-ACTIN monoclonal antibody (1:3000, HRP-60008, ProteinTech), mouse anti-α-TUBULIN monoclonal antibody (1:10000, T5168, Sigma-Aldrich), HRP-conjugated goat anti-mouse IgG polyclonal antibodies (1:10000, 31430, Invitrogen) and HRP-conjugated goat anti-rabbit IgG polyclonal antibodies (1:10000, 31460, Invitrogen).

### Cell cycle analysis

The cells were harvested and preserved by immersing them in ice-cold 70% (v/v) ethanol for 24 h at 4 °C. Following this, the cells underwent staining with a solution comprising RNase A (10 µg/mL, Geneaid) and propidium iodide (50 µg/mL, P4170, Sigma-Aldrich) in PBS containing 0.5% Triton X-100 for 30 min in darkness. DNA content was measured using the Beckman Coulter FC500 MCL System with CXP software, with the parameter set to FL3-620 nm BP. Approximately 10,000 events were collected for each sample, and the data were analysed using the CXP software.

### Immunohistochemistry

Frozen tissue sections were treated with 0.3% H_2_O_2_ for 5 min and then blocked with protein blocking buffer (ab64226, Abcam). After washing with PBS, the samples were incubated with primary antibodies in PBS with 2.5% BSA and 0.25% Triton X-100 for at least 2 h at room temperature. The first antibodies used were rabbit anti-gamma H2A.X (phospho S139) polyclonal antibody (γH2AX, ab2893, Abcam). After washing with PBS, the samples were incubated with biotinylated goat anti-rabbit IgG antibody (BA-1000, Vector Laboratories) for at least 2 h followed by another PBS wash. All tissue staining was performed in parallel with control sections stained with only secondary antibodies. Antibodies were applied at a 1:500 dilution. To detect the secondary antibody, the VECTASTAIN ABC kit (PK-6100, Vector Laboratories) and DAB Quanto Chromogen and Substrate (TA-125-QHDX, Invitrogen) were used following the manufacturer’s instructions.

### Blastocoel injection

Eight-week-old female ICR mice were superovulated by peritoneal injection of 5 IU of pregnant mare serum gonadotropin (PMSG) followed by 5 IU of hCG 48 h later to stimulate egg production. Blastocyst-stage embryos were obtained from the uterus horn of superovulated females that had been mated with ICR males 94–98 h post-hCG. These blastocysts were injected with 15–20 ESCs using a pulled micro glass needle, and then transferred into the uteri of 2.5 days post coitum pseudopregnant ICR females that had been mated with vasectomised males. The resulting chimeras derived from blastocyst injection were obtained by natural birth.

### Statistical analysis

The statistical analysis was conducted by GraphPad Prism software through Student’s t-test or one-way ANOVA. Manual counting and quantification of nuclei, cytoophidia, as well as EdU-labelled and p-H3-positive cells were performed using Fiji software for image analysis. The IHC profiler plugin in Fiji software was utilised to determine the staining intensity of γH2AX. A comprehensive dataset was compiled from a minimum of three independent experiments, with each quantification involving the examination of over 100 cells. Standard error of the mean (SEM) was employed for the representation of error bars in all graphical illustrations. Western blot experiments were independently carried out at least three times, and band intensity, measured using Fiji software, was normalised to the housekeeping reference.

## Results

### Cytoophidium assembly is correlated with active glycolysis and PPP in mouse PSCs

While IMPDH cytoophidia can be induced by specific drugs or culture conditions lacking certain nutrients like glutamine, these filamentous structures are also naturally present in certain tissues and cultured cells [6, 16, 22, 23]. For example, over 80% of mouse ESCs and iPSCs exhibit IMPDH cytoophidia under standard culture conditions [11, 16]. However, induction of cell differentiation by retinoic acid (RA) in mouse ESCs leads to a dramatic loss of IMPDH cytoophidia, implying a correlation between cytoophidium regulation and the unique features of ESCs [16].

In contrast to differentiated cells, rapidly dividing PSCs display a heightened dependence on glycolysis. This metabolic preference shares similarities with the aerobic glycolysis observed in cancer cells and plays a pivotal role in sustaining the stemness of PSCs. The glycolytic metabolism not only provides energy source but also supports biomass generation, including nucleotides that are essential for cell proliferation [24]. Thus, we aimed to investigate deeper into the relationship between IMPDH cytoophidium regulation and the metabolic shift during PSC differentiation.

**Fig. 1:**
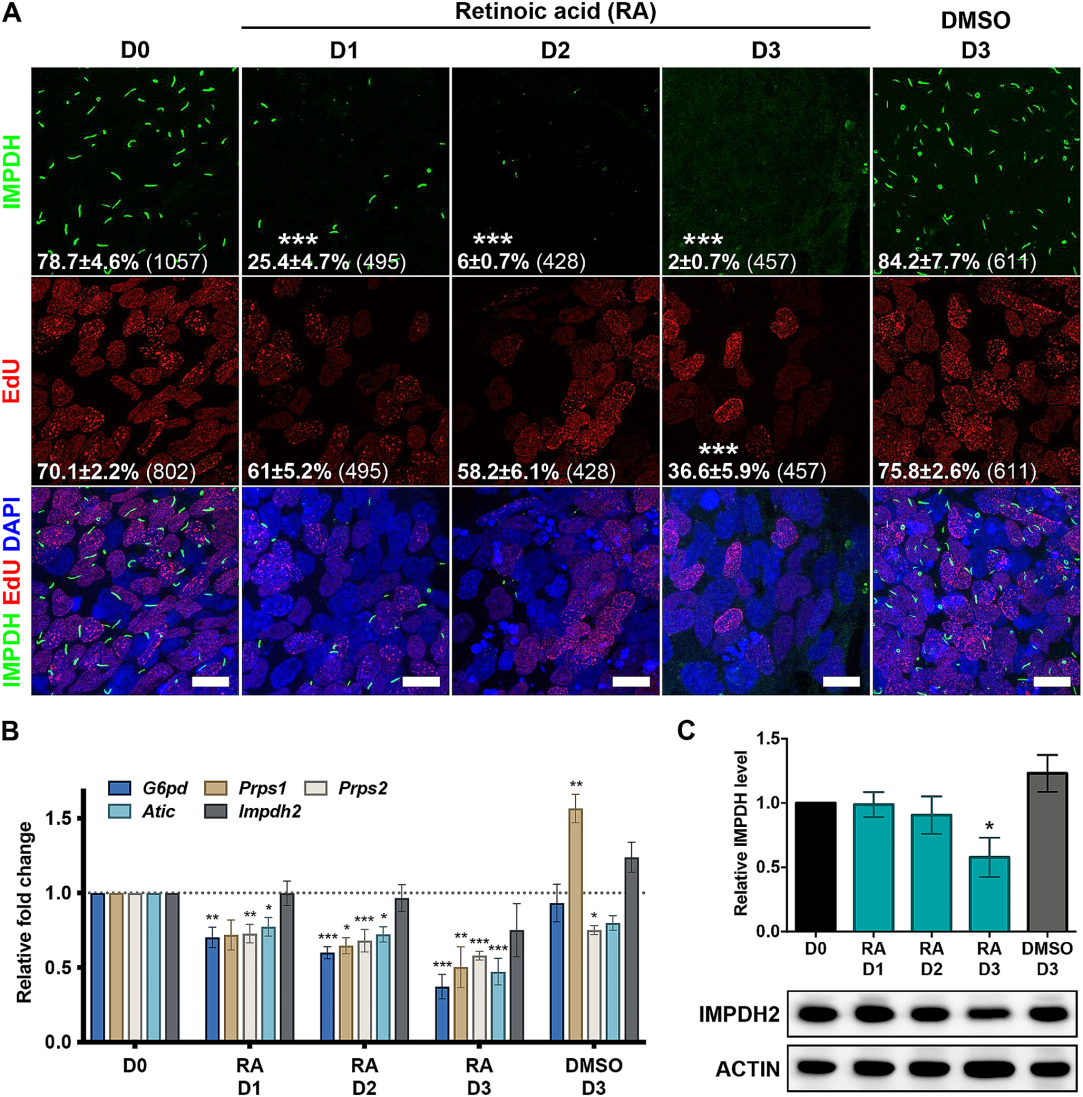
IMPDH cytoophidia disassemble during mouse iPSC differentiation. (**A**) Immunofluorescence analysis of IMPDH (green) in mouse iPSCs treated with RA for 0–3 days. Proliferating cells are labelled with EdU (red). Mean percentage and SEM of cells with cytoophidia and EdU labelling are presented in the lower-left corner. Total cell counts from at least three independent experiments are indicated in brackets. DAPI staining is shown in blue, and scale bars represent 10 μm. (**B**) Gene expression levels of key enzymes involved in the first step of the PPP (*G6pd*), as well as enzymes upstream of *de novo* nucleotide biosynthesis (*Prps1/2* and *Atic*) and *Impdh2*, are measured in iPSC treated with RA. (**C**) Western blot and quantitation of IMPDH protein normalised to ACTIN for iPSCs treated with RA. Groups are compared by one-way ANOVA with D0 and error bars represent SEM (*P < 0.05, **P < 0.01, ***P < 0.001). In (**B**) and (**C**), each bar represents the average relative fold change compared to the control group D0 in each replicate

First, we induced iPSCs differentiation using 2 µM RA for 1 to 3 days (Fig. 1A). The proportion of cells with cytoophidia rapidly decreased from 79 to 25% within the first day of RA treatment, further dropping to less than 10% thereafter (Fig. 1A). The proliferation rate, as indicated by ethynyl deoxyuridine (EdU) labelling, exhibited a slight decline from 70% to around 60% on days 1 and 2, followed by a more significant reduction to 37% on day 3 (Fig. 1A).

Concurrently, on the first day of cell differentiation, the expression levels of key genes involved in the PPP and nucleotide synthetic pathway, including *G6pd*, *Prps* and *Atic*, decreased to around 70% of their initial levels, gradually reducing to approximately 50% by day 3 (Fig. 1B). Meanwhile, the levels of IMPDH protein also declined to around 50% on day 3 (Fig. 1C). These findings suggest that alterations in the molecular flux within the *de novo* nucleotide synthesis pathway manifest as early as the first day of cell differentiation, concurrent with IMPDH cytoophidium disassembly.

**Fig. 2:**
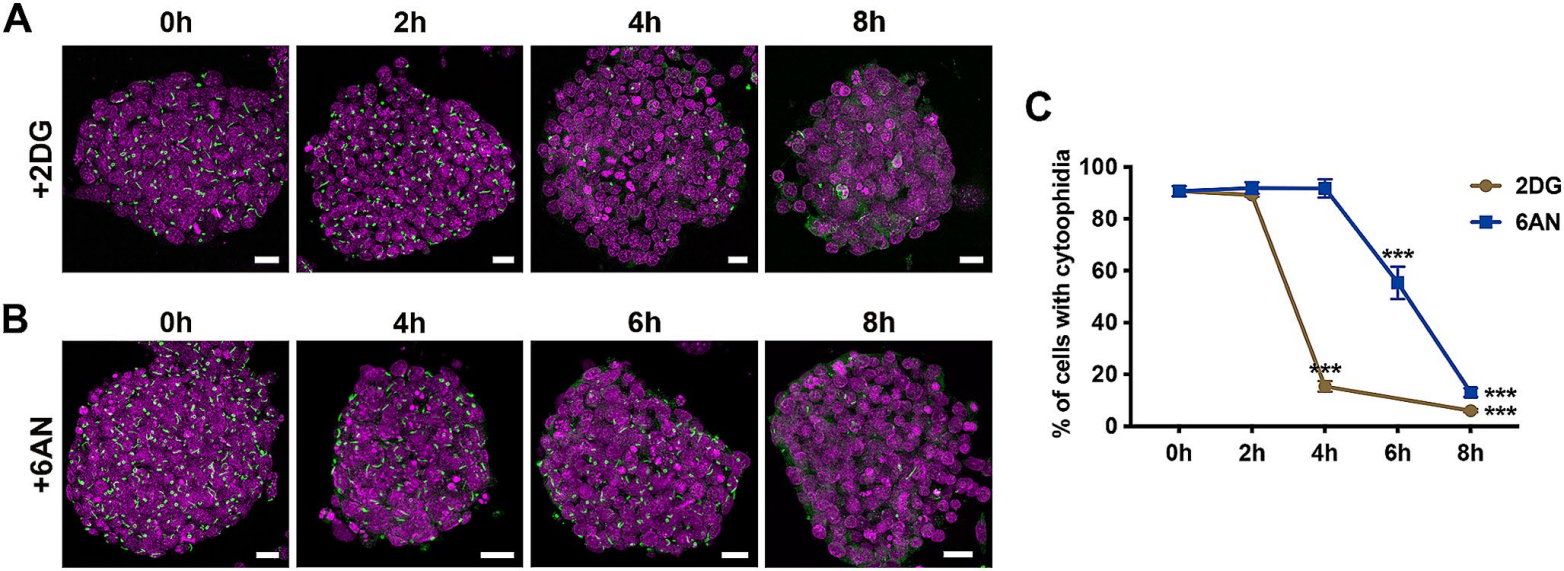
IMPDH cytoophidia disassemble in mouse ESCs upon inhibition of glycolysis and PPP. (**A** and **B**) Mouse ESCs are treated with (**A**) 2DG, a glycolysis inhibitor, or (**B**) 6AN, a PPP inhibitor, for 0–8 h, and probed with an anti-IMPDH antibody (green). DAPI staining is shown in magenta, and scale bars are 20 μm. (**C**) Quantitative analysis of the percentage of cells with cytoophidia under different treatment conditions in (**A** and **B**). Groups are compared by one-way ANOVA with 0 h and error bars represent SEM (***P < 0.001)

We then investigated whether inhibiting glycolysis or the PPP would trigger the disassembly of IMPDH cytoophidium in mouse PSCs. Mouse iPSCs were treated with 10 mM 2-deoxyglucose (2DG), a hexokinase (HK) inhibitor, and 200 µM 6-aminonicotinamide (6AN), a G6PD inhibitor, to inhibit glycolysis and the PPP, respectively (Fig. 2A and B). Following a 4-hour inhibition of glycolytic metabolism, the proportion of cells with cytoophidia dramatically decreased from 90 to 15%, further reducing to 6% after 8 h (Fig. 2A and C). In contrast, a clear decline in IMPDH cytoophidia in cells with PPP inhibition was not observed until 6 h of treatment, dropping from 90 to 55%. By 8 h, cytoophidia were present in only 13% of cells (Fig. 2B and C). These results indicate a correlation between active glycolysis and PPP and the formation of IMPDH cytoophidium in mouse PSCs.

### The formation of IMPDH cytoophidium corresponds with metabolic changes rather than pluripotency during somatic cell reprogramming

During somatic reprogramming for iPSC generation, there is a notable transition from oxidative phosphorylation to glycolysis [25]. The upregulation of glycolytic genes occurs early in the reprogramming process, preceding the induction of pluripotency genes and remaining upregulated throughout the reprogramming process. Increased glycolytic activity enhances reprogramming efficiency, while inhibition disrupts the process [24, 26]. This metabolic shift during cellular reprogramming presents a contrasting model to PSC differentiation, offering insight into the correlation between cytoophidium assembly and PSC metabolism.

**Fig. 3:**
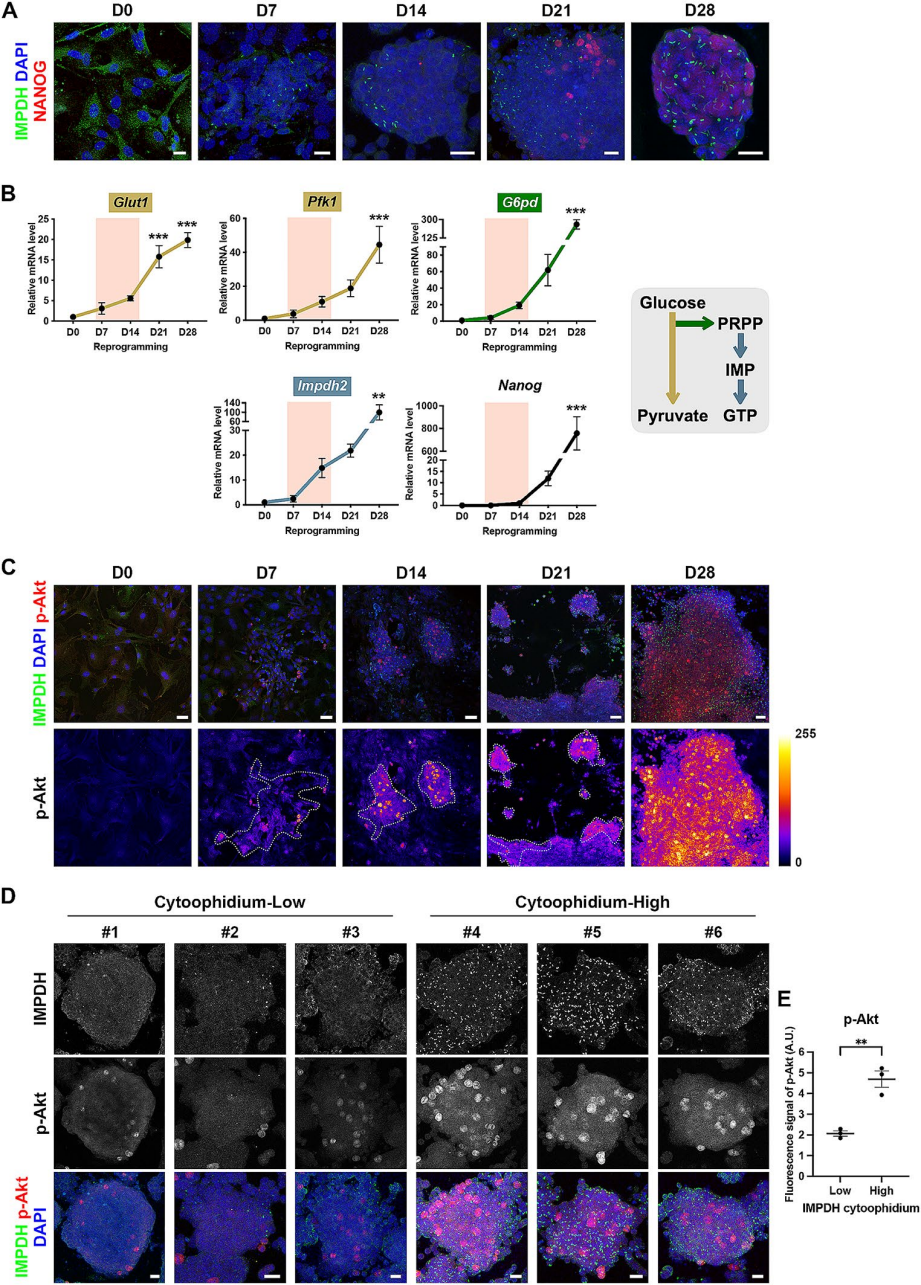
Cytoophidium assembly is correlated with the upregulation of glycolysis during mouse embryonic fibroblast reprogramming. (**A**) Immunofluorescence of IMPDH (green) and pluripotent marker NANOG (red) in cells at different time points after reprogramming induction. D stands for day. (**B**) Gene expression levels of glycolytic enzymes (*Glut1* and *Pfk1*, shown in tan), the rate-limiting enzyme in the PPP (*G6pd*, shown in green), *Impdh2* (shown in blue) and pluripotent marker (*Nanog*) at different time points after reprogramming induction. The expression of cytoophidia and *Impdh* mRNA is accompanied by the upregulation of glycolysis-related genes but not pluripotency. (**C** and **D**) Immunofluorescence of IMPDH (green) and p-Akt (red) in (**C**) cells at different time points after reprogramming and in (**D**) colonies selected after 28 days of reprogramming. The Fire Lookup Table, applied using Fiji software, illustrates the fluorescence intensity of p-Akt in (**C**). Dashed white lines outline areas with numerous cytoophidia in the images of D7-21 reprogramming cells. In (**D**), colonies #1–3 represent those with few cells exhibiting cytoophidia (cytoophidium-low), while colonies #4–6 represent those with many cytoophidia (cytoophidium-high). DAPI staining is shown in blue, with scale bars of 20 μm in (**A** and **D**) and 50 μm in (**C**). (**E**) The scatter plot illustrates the mean fluorescence intensity of p-Akt in (**D**). Statistical significance is evaluated using one-way ANOVA (with each group compared with D0) in (**B**) and Student’s t-test in (**E**). Error bars represent SEM (**P < 0.01, ***P < 0.001)

To induce cellular reprogramming, mouse embryonic fibroblasts were transduced with retrovirus carrying Yamanaka factors (POU5F1/OCT4, SOX2, KLF4 and c-Myc). Cells collected at various time points post-infection, including day 0 (representing somatic cells), day 7 and day 14 (transitional cells), day 21 (early-stage iPSCs) and day 28 (fully reprogrammed iPSCs), for analysis. Intriguingly, during days 7 to 14 of the reprogramming, we could already observe IMPDH cytoophidia in some aggregating cells (Fig. 3A). At this stage, *Impdh* mRNA was elevated, along with increased expression of glycolysis-related genes such as *Glut1* and *Pfk*, as well as the PPP gene *G6pd*, while the pluripotent marker *Nanog* had not yet been induced (Fig. 3A and B).

Furthermore, the presence of IMPDH cytoophidium exhibited a correlation with the activation of Akt (phosphorylated at S473, p-Akt) during the reprogramming process (Fig. 3C). Akt is known for its essential role in maintaining the undifferentiated state of ESCs and upregulating glycolytic genes during somatic cell reprogramming [27]. Notably, colonies with fewer IMPDH cytoophidia displayed significantly lower immunofluorescent signals for p-Akt compared to colonies with abundant cytoophidia, illustrating a positive correlation between cytoophidium abundance and Akt activity in PSC colonies (Fig. 3D and E). These findings provide additional evidence for the association between cytoophidium assembly and metabolic features of PSCs.

### Establishment of no-cytoophidium mutant mouse ESC lines

In iPSCs, supplementation of guanosine or cell cycle arrest leads to IMPDH cytoophidium disassembly, which reassembles upon guanosine removal and cell cycle resumption. These observations demonstrate the role of the cytoophidium in maintaining the GTP pool during rapid cell proliferation [11]. Despite various conditions known to affect cytoophidium assembly and disassembly, it is still challenging to determine the function and importance of IMPDH polymerisation and cytoophidium assembly without a genetical manipulation specifically targeting this mechanism.

**Fig. 4:**
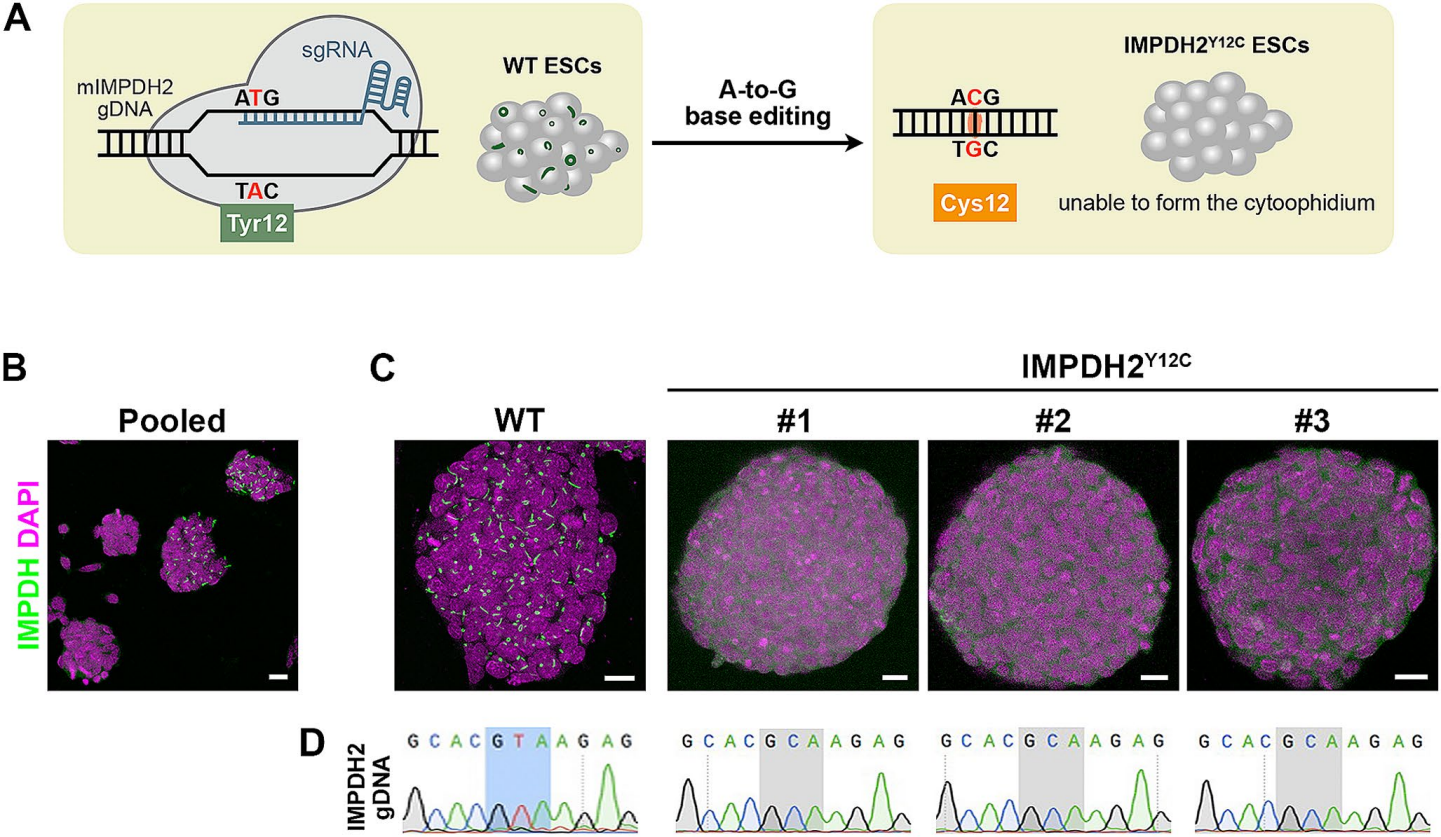
Generation of mutant mouse ESCs with no-IMPDH cytoophidium phenotype. (**A**) Schematic representation of the generation of IMPDH2^Y12C^ mutant cell line using ABE-mediated base editing. (**B**) After base editing, a heterogeneous population of cells is obtained. Cells are probed with an anti-IMPDH antibody shown in green. Single cell-derived colonies are manually picked for (**C**) immunofluorescence analysis of IMPDH (green) and (**D**) sequencing of the targeted DNA region. Three mutant ESC lines with a no-cytoophidium phenotype are identified. WT stands for wild type. DAPI staining is shown in magenta, and scale bars represent 20 μm

Previous studies have revealed that disrupting the IMPDH polymer interface with a point mutation, Y12A, abolishes IMPDH polymerisation without affecting its catalytic machinery [7, 10]. This makes Y12 a promising target for establishing a model to explore the functions of IMPDH polymers and the cytoophidium. In our parallel study, we show efficient introduction of the Y12C point mutation into human IMPDH1 and IMPDH2 genomic sequences using a dCas9-based adenine base editor, ABEmax. The IMPDH2 Y12C mutation does not induce significant cell toxicity or growth defects in cancer cell lines in vitro [21]. Therefore, we aimed to employ this strategy to establish genetically modified mouse ESCs (Fig. 4A).

Mouse ESCs were transfected with two constructs: one encoding ABEmax with an mCherry reporter and another encoding sgRNA targeting mouse IMPDH2 Y12. Two days post-transfection, mCherry-positive cells were sorted using a cell sorter and cultured in the dish as a heterogeneous population (Fig. 4B). After dissociating colonies into single cells and further subculturing, grown colonies were manually selected and screened through immunofluorescence. Out of 24 colonies, 5 exhibited a no-cytoophidium phenotype. Among them, 3 ESC lines were randomly chosen and confirmed by Sanger sequencing to have undergone successful IMPDH2 Y12C genomic editing (Fig. 4C and D).

### The loss of cytoophidium leads to downregulated expression of genes in glycolysis, PPP and purine nucleotide synthesis

**Fig. 5:**
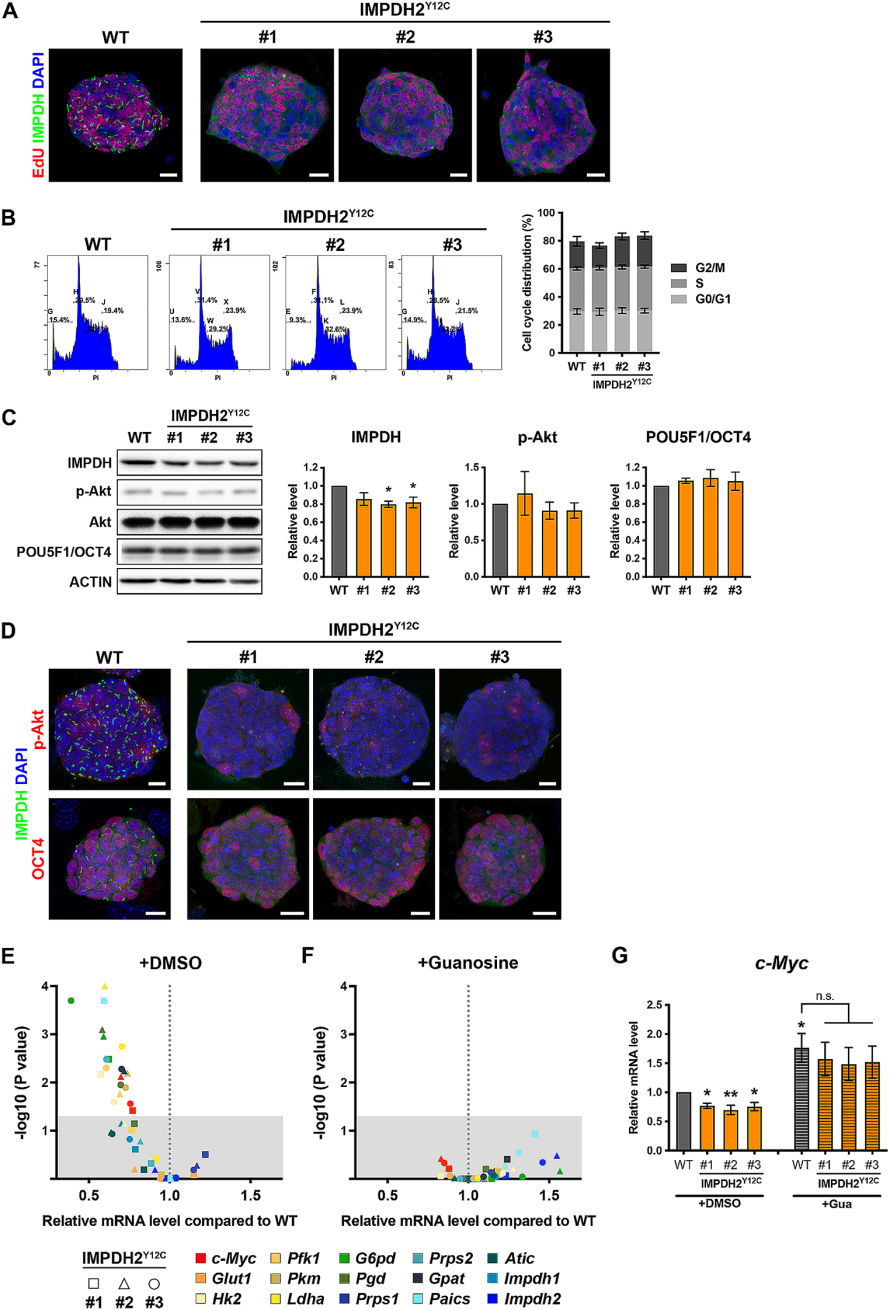
Cytoophidium assembly is important for maintaining the expression of genes involved in glycolysis-derived nucleotide synthesis in mouse ESCs. (**A**) Immunofluorescence of IMPDH (green) and proliferating cells labelled by EdU (red) in WT and mutant ESCs. (**B**) Flow cytometry analysis and quantification for the DNA content in WT and mutant ESCs. (**C**) Western blot and quantification of IMPDH and POU5F1/OCT4 normalised to ACTIN, as well as Akt in WT and IMPDH2^Y12C^ ESCs. (**D**) Immunofluorescence of IMPDH (green) and p-Akt (red in upper panels), or pluripotent marker POU5F1/OCT4 (red in lower panels) in WT and mutant ESCs. DAPI staining is shown in blue, and scale bars are 20 μm. (**E** and **F**) The scatter plots show the relative mRNA levels of *c-Myc* and genes involved in glycolysis-derived nucleotide synthesis in mutant ESCs compared to WT cells cultured under media (**E**) with or (**F**) without guanosine (Gua) supplementation, plotted against the corresponding *p*-values. The shaded region indicates *p*-values greater than 0.05. (**G**) Gene expression levels of *c-Myc* in WT and mutant ESCs cultured under media with or without guanosine supplementation. In (**C** and **G**), grey bars represent WT, and the orange bars represent mutant ESCs. Bars in (**G**) that are filled with lines indicate the condition with guanosine supplementation. Groups without highlighting are compared with WT ESCs cultured under normal conditions, and error bars represent SEM (one-way ANOVA, *P < 0.05, **P < 0.01)

Although the mutant ESCs are incapable of assembling IMPDH cytoophidium, we observed no significant difference in cell growth assessed by EdU labelling and cell cycle stages determined by flow cytometry (Fig. 5A and B). Our next goal was to investigate the effects of IMPDH2 Y12C mutation on ESC metabolic regulation.

In our previous study, we have shown that forming the cytoophidium may protect the protein from degradation [9]. In IMPDH2^Y12C^ mutant ESC lines, we found a mild decrease in IMPDH protein levels by approximately 20% in two mutant ESC lines (Fig. 5C). Although the antibody used in this study specifically targets the IMPDH2 protein, the considerable 84% similarity between IMPDH1 and IMPDH2 may result in cross-reactivity of the antibody with both isoforms. Consequently, it is plausible that the observed 20% decrease in IMPDH protein expression could also be attributable to differences in *Impdh1* levels rather than *Impdh2*. On the other hand, several studies have established the pivotal role of PI3K/Akt signalling in regulating ESC self-renewal, pluripotency and somatic cell reprogramming. Disrupting this pathway has been revealed to reduce the expression of pluripotency markers and impair ESC self-renewal capacity [28, 29]. However, all IMPDH2^Y12C^ mutant ESC lines displayed similar levels of p-Akt and comparable expression of pluripotency marker POU5F1/OCT4 compared to wildtype cells (Fig. 5C and D).

Next, we performed real-time PCR analysis to examine the expression of key genes involved in glycolysis (*Glut1*, *Hk2*, *Pfk1*, *Pkm* and *Ldha*), PPP (*G6pd* and *Pgd*) and purine synthesis (*Prps1/2*, *Gpat*, *Paics*, *Atic* and *Impdh1/2*). Most genes showed a notable reduction in expression across all mutant ESC lines, except for *Glut1*, *Prps1* and *Impdh2* (Fig. 5E and F). Phosphofructokinase-1 (*Pfk1*), the rate-limiting enzyme of glycolysis, is critical for determining glycolytic flux. Downregulation of the expression of PFK1 has been shown to decrease the glycolytic flux in certain cell types [30, 31]. Glucose-6-phosphate dehydrogenase (*G6pd*), the rate-limiting enzyme of PPP, plays a similar pivotal role in PPP. The inhibition or downregulation of G6PD may reduce PPP flux and induce cell death [32, 33]. In addition, the expression level of the oncogene c-Myc, a well-known regulator of these metabolic pathways, was lower in mutant ESCs (Fig. 5E-G). Interestingly, when the cells were cultured in a medium supplemented with 100 µM guanosine, which fuels intracellular GTP levels through the salvage pathway, the expression levels of *c-Myc* and all metabolic enzymes mentioned above restored to those in wildtype cells (Fig. 5E-G). These findings suggest that the loss of IMPDH polymers and the cytoophidium does significantly alter the regulation of cell proliferation and pluripotency but may induce changes in the metabolic profile of ESCs. This could be attributed to a decrease in GTP concentration in mutant ESCs, although an unexpected function of IMPDH polymerisation may also contribute to the regulation of gene expression.

### The loss of the cytoophidium and associated metabolic changes attenuate teratoma growth in vivo

In cell culture, cells are typically supplied with sufficient nutrients through the medium and supplements. We hypothesised that the nutrient-rich culture medium might mask potential defects resulting from the IMPDH mutation. To further explore the impacts of the IMPDH2 Y12C mutation, we conducted a teratoma formation assay to evaluate in vivo growth differences between wildtype and mutant ESCs.

**Fig. 6:**
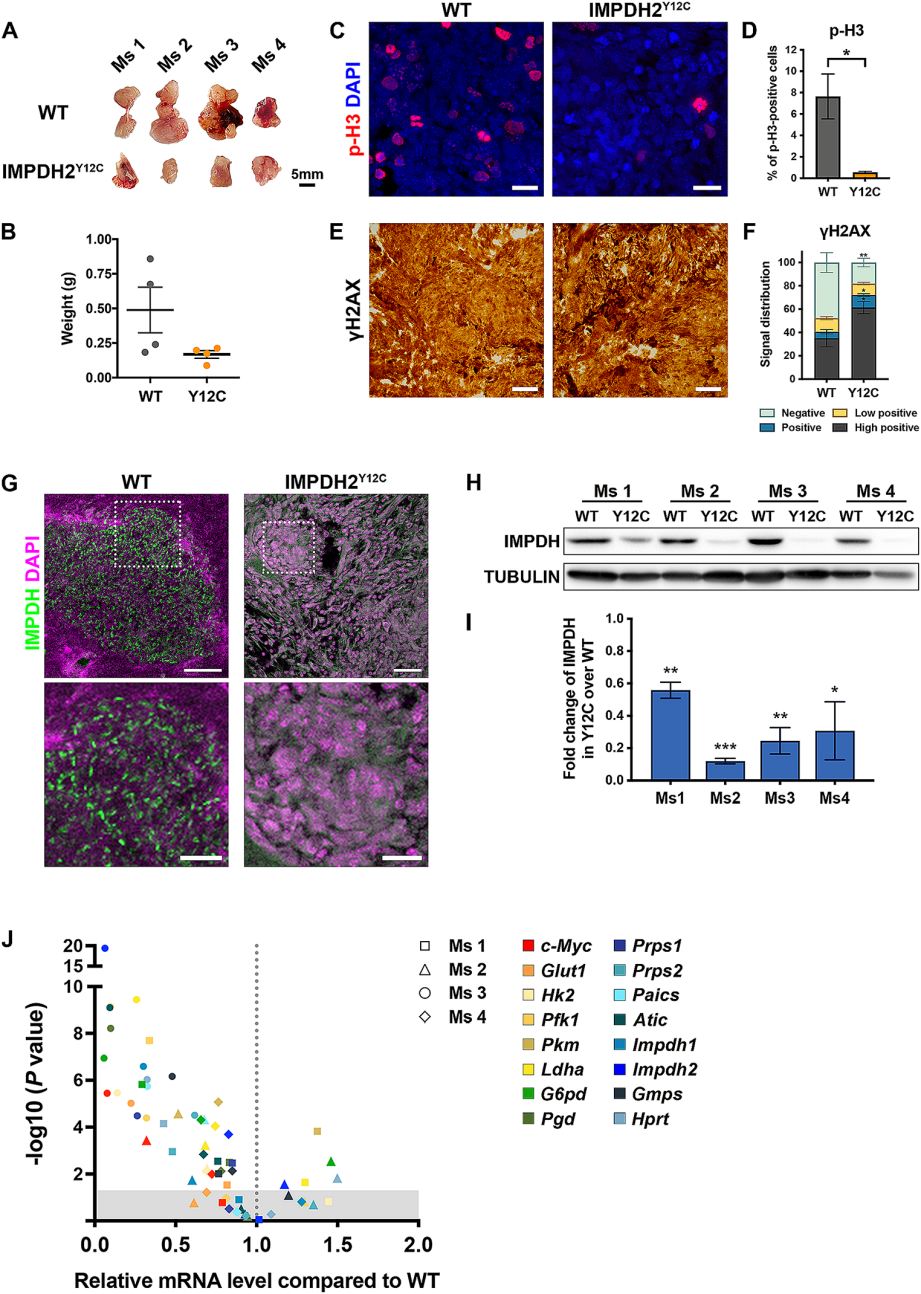
The loss of IMPDH cytoophidium alters gene expression in ESC-derived teratomas. (**A** and **B**) Morphology and weight of teratomas derived from WT and IMPDH2^Y12C^ ESCs in 4 immune-deficient mice (Ms). Scale bar is 5 mm. (**C**) Immunofluorescence of p-H3 (red), a proliferation marker, in teratoma sections. DAPI staining is shown in blue, and scale bars are 20 μm. (**D**) The percentage of p-H3-positive cells is lower in mutant tissues. (**E**) Immunohistochemical analysis of γH2AX, a DNA damage marker, in teratoma sections. Scale bars are 100 μm. (**F**) The expression levels of γH2AX are classified into high positive, positive, low positive and negative groups using the IHC profiler plugin in Fiji software. The mutant group exhibits significantly higher percentages of cells with high positive and positive γH2AX signals, along with significantly lower percentages of cells with negative signals compared to the WT group. (**G**) Teratoma sections stained with IMPDH (green) and DAPI (magenta) showing cytoophidia in WT but not mutant tissues. Magnified images of dashed white boxes are shown below. Scale bars are 50 μm (top) and 20 μm (bottom). (**H** and **I**) Western blot and quantification of IMPDH normalised to TUBULIN in teratomas. (**J**) The scatter plot shows the relative mRNA levels of *c-Myc* and genes involved in glycolysis-derived nucleotide synthesis in mutant compared to WT teratomas derived from four mice, plotted against the corresponding *p*-values. Data are presented as fold change of WT over mutant in (**I** and **J**). Statistical significance is evaluated using Student’s t-test to compare WT and mutant samples. The shaded region in (**J**) indicates *p*-values greater than 0.05. Error bars represent SEM (*P < 0.05, **P < 0.01, ***P < 0.001)

Injecting ESCs into the thigh muscles of immunodeficient mice resulted in teratoma formation by both wildtype and IMPDH2^Y12C^ ESCs. After 5 weeks, we harvested the transplants and found that teratomas from mutant ESCs in 2 out of the 4 hosts exhibited smaller sizes compared to their wildtype counterparts (Fig. 6A and B). We also examined histone H3 phosphorylation on serine 10 (p-H3) and histone H2AX phosphorylation on serine 139 (γH2AX), which are markers for mitosis and DNA damage, respectively [34, 35]. The results show a smaller proportion of cells expressing p-H3 and a greater proportion of cells expressing high levels of γH2AX in mutant tissues. This indicates reduced proliferation and increased DNA disability, which may lead to heightened apoptotic potential and cell death (Fig. 6C-F).

Immunofluorescence analysis of tissue sections reveals the widespread presence of IMPDH cytoophidia in all wildtype transplants but not in the mutant teratomas (Fig. 6G). Moreover, the levels of IMPDH protein were significantly lower in mutant tissues (Fig. 6H and I). Real-time PCR analysis indicates a general decrease in the expression of *c-Myc* and genes related to glycolysis, PPP and purine nucleotide synthesis in mutant teratomas, consistent with the results observed in cultured ESCs (Figs. 5E and F and 6J). These data suggest that alterations in the expression of genes in metabolic pathways, associated with the IMPDH Y12C mutation, may lead to metabolic imbalance and eventually render growth defects in vivo.

### IMPDH cytoophidium assembly may participate in the regulation of cell metabolism during mouse fetal development

**Fig. 7:**
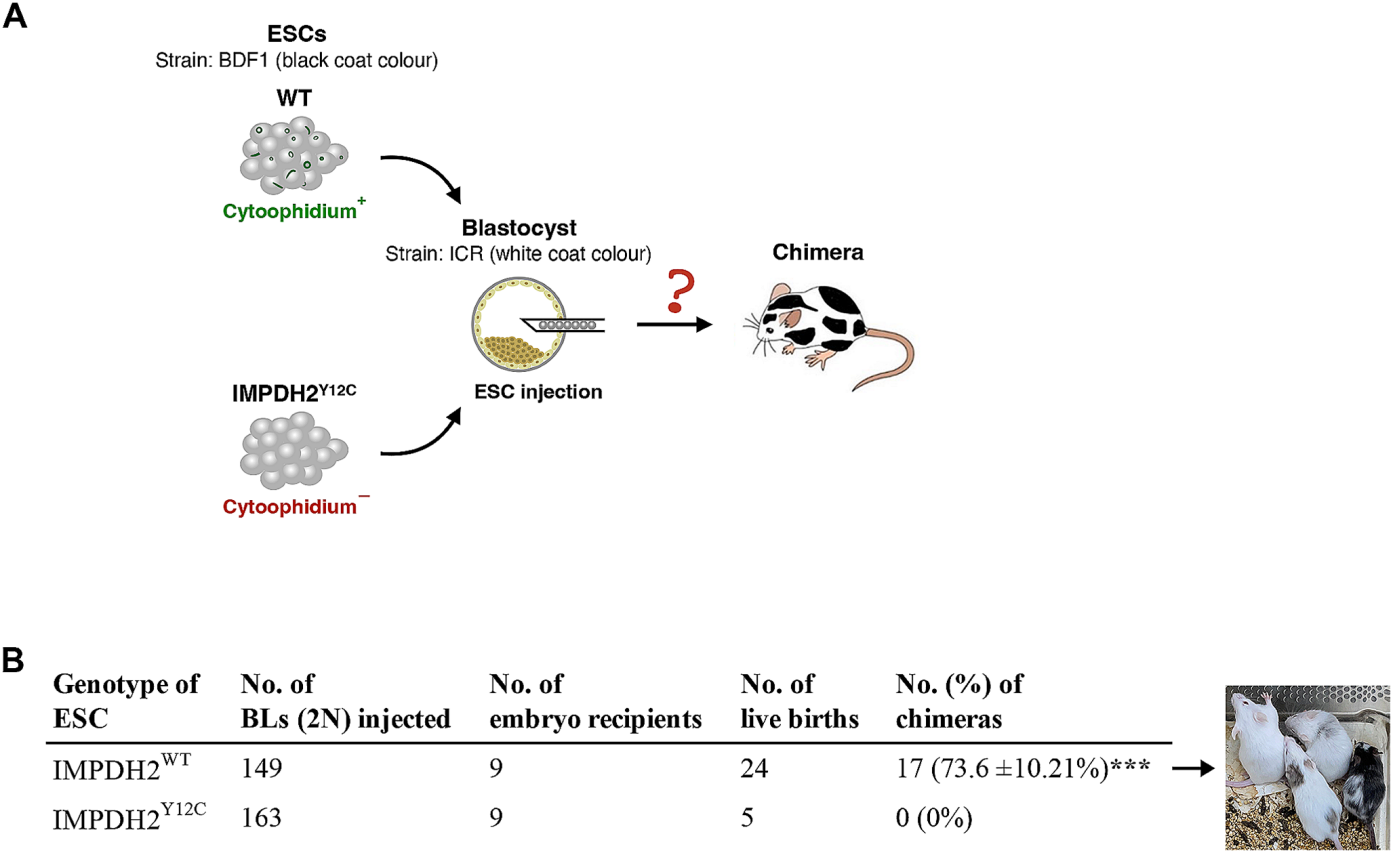
Absence of IMPDH cytoophidium impairs chimeric mouse generation by ESCs. (**A**) Schematic illustrating the production of chimeric mice through the microinjection of BDF1 ESCs into the cavity of a blastocyst derived from ICR mice. Given the contrasting coat colours of BDF1 (black) and ICR (white) mice, the resulting chimeras display a mixture of black and white fur, serving as an indicator of the participation of injected ESCs in fetal development following blastocyst injection. (**B**) Quantification and representative image of chimeras obtained after blastocyst injection with WT and IMPDH2^Y12C^ ESCs. Statistical significance is evaluated using Student’s t-test, and error bars represent SEM (***P < 0.001)

To assess the impact of disrupting IMPDH polymerisation and cytoophidium assembly on ESC pluripotency, we performed blastocoel microinjection using two mutant ESC lines (line #1 and #2) and wildtype ESCs. Microinjection of stem cells into blastocysts is a commonly used technique to generate chimeras and investigate their contribution to embryonic development. The ESCs utilised in this study were derived from B6D2F1 (BDF1) mice, characterised by a black coat colour. When these ESCs are injected into blastocysts obtained from Crl: CD1(ICR) mice, which have white coat colour, chimeras displaying a mixture of white and black fur are easily identifiable (Fig. 7A). Out of 149 embryos injected with wildtype ESCs and transferred into surrogate mothers, 24 embryos developed to live pups. Among them, 17 (73.6%) were identified as chimeric based on their coat colour (Fig. 7B). Surprisingly, in 163 embryos injected with mutant ESCs, only 5 mice were born. Furthermore, none of these live births exhibited chimerism, indicating impaired fetal development involving the integration of mutant ESCs (Fig. 7B).

The developmental defects observed in IMPDH2^Y12C^ ESCs-injected embryos highlight an indispensable role of IMPDH polymerisation in embryonic or fetal development. Previous studies have demonstrated that IMPDH expression rises and peaks during the morula and early blastocyst stages [36]. Inhibition of IMPDH, either by inhibitors or genetic knockout, disrupts embryo development up to the blastocyst [2, 37]. However, in our model the abnormal development resulting from the IMPDH2 mutation must have occurred after the blastocyst stage, implying that IMPDH2 polymerisation might be crucial for post-implantation development as well. Indeed, the nucleotide pool is rapidly and continuously generated from glucose via the PPP starting from embryonic day (E) 10.5 [38]. These findings prompted us to investigate the spatiotemporal regulation of IMPDH cytoophidium assembly.

**Fig. 8:**
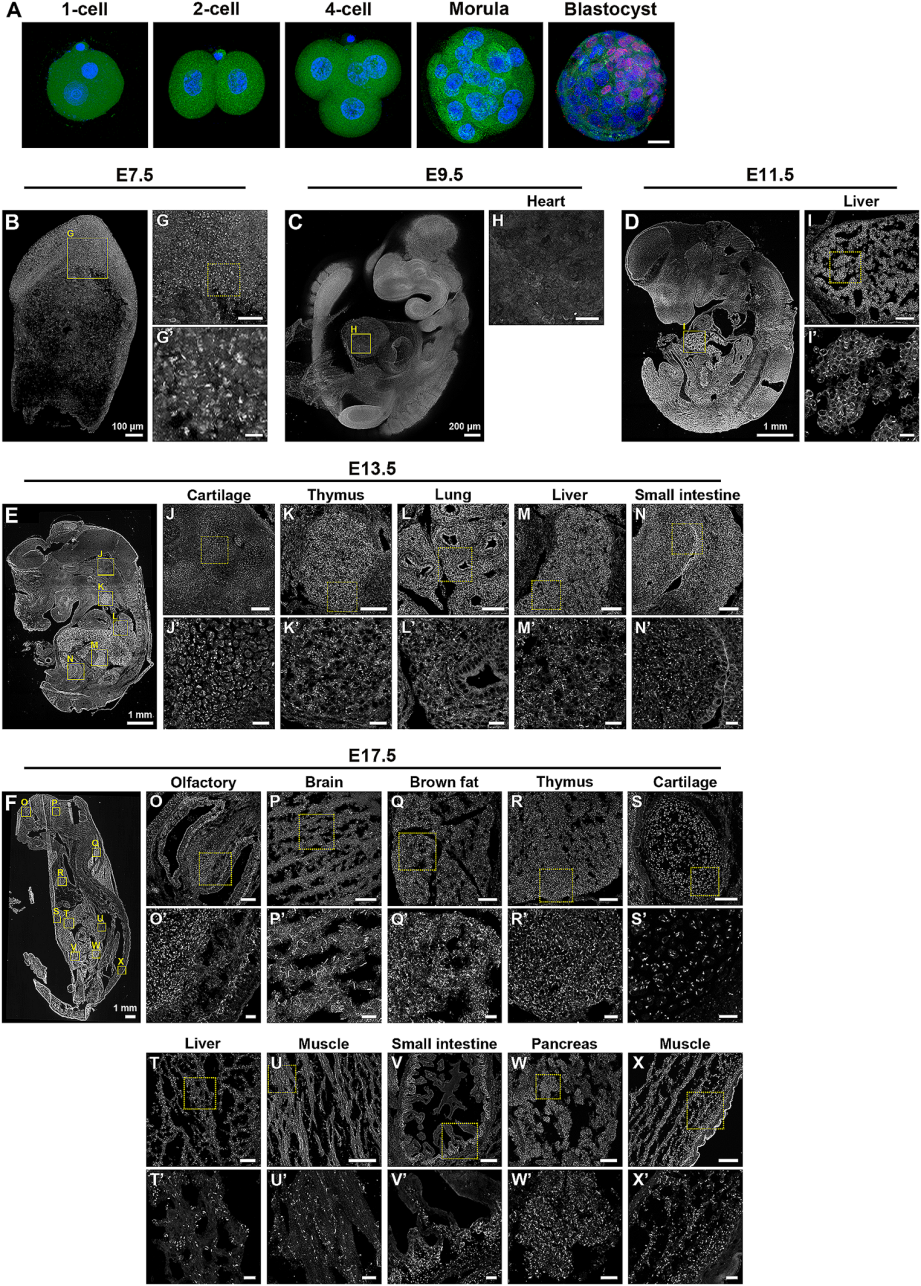
IMPDH forms cytoophidia during mouse embryo development. (**A**) Immunofluorescence of IMPDH (green) in mouse pre-implantation embryos, with the location of the inner cell mass in the blastocyst marked by anti-POU5F1/OCT4 antibody (red). DAPI is shown in blue, and the scale bar is 20 µm. (**B**-**F**) Immunofluorescence of IMPDH in mouse embryos at E7.5, E9.5, E11.5, E13.5 and E17.5. (**G**-**X**) magnify selected areas in (**B**-**F**), and (**G’**-**X’**) are further magnified views of selected areas in (**G**-**X**). Scale bars are 100 μm in (**B**), 200 μm in (**C**), 1 mm in (**D**-**F**), 50 μm in (**G** and **H**), 10 μm in (**G’**), 100 μm in (**I**-**X**) and 20 μm in (**I’**-**X’**)

Mouse embryos and fetuses were collected at various developmental stages and subjected to immunofluorescence analysis for the presence of IMPDH cytoophidium. While cytoophidia were rarely detected in pre-implantation stage embryos, many IMPDH cytoophidia were found in the tissues of E7.5 embryos (Fig. 8A and B). Intriguingly, IMPDH cytoophidia diminished in all tissues at E9.5 and reappeared in the liver at E11.5 (Fig. 8C and D). Later on, IMPDH cytoophidia were also detected in other tissues such as cartilage, thymus, lung and small intestines at E13.5, as determined by tissue morphology (Fig. 8E).

At a more advanced fetal stage (E17.5), an increased amount of IMPDH cytoophidia was observed throughout the entire fetus (Fig. 8F). The gradual increase in the abundance of IMPDH cytoophidia over fetal development provides further evidence for the crucial roles of IMPDH cytoophidium in coupling cell metabolism and development. Altogether, our results offer new insights into the essential functions of IMPDH polymerisation and cytoophidium assembly in embryogenesis and fetal development.

## Discussion

The polymerisation of IMPDH has been demonstrated to desensitise IMPDH from GTP feedback inhibition in vitro and has been proposed to enhance the flux of GTP production through the *de novo* GMP synthesis pathway in the cell [6, 7]. In addition, the cytoophidium structure has been shown to protect IMPDH from degradation, providing an additional boost to purine synthesis.

The assembly of the cytoophidium by IMPDH polymers could be facilitated by an increase in molecular crowding within the cell [9]. Both cytoplasmic crowding and cytoophidium assembly have been shown to be positively correlated with mTORC1 activity and cell proliferation [6, 19]. It is reasonable to speculate that IMPDH polymerisation and cytoophidium assembly may function to couple cell proliferation with cell metabolism.

In fact, previous studies have revealed that IMPDH cytoophidia are commonly present in rapidly proliferating cells, such as mouse PSCs, activated T cells and developing thymocytes [11–13, 15, 16]. Despite these correlational studies, the physiological significance of IMPDH cytoophidium remains unclear due to challenges in disrupting IMPDH polymerisation without extreme manipulations or introducing extra proteins into the cell. In this study, we proved that the CRISPR/base editing-based strategy can effectively and precisely disrupt IMPDH polymerisation in the cells.

We have previously demonstrated that deleting as few as 6 residues in the Bateman domain of IMPDH2 is sufficient to prevent cytoophidium formation in HeLa cells [11]. Under normal culture conditions, no growth defects were observed in IMPDH mutant cells. However, cell proliferation was significantly suppressed in IMPDH mutant HeLa cells when IMPDH expression was knocked down. In contrast, wildtype cells, which displayed many cytoophidia under the same conditions, remained normal proliferation rate [11].

Based on these results, we proposed that the cytoophidium is required for cells to endure certain metabolic stress. Consistently, we did not find notable defects in cell proliferation or cell death in the in cultured IMPDH2^Y12C^ ESCs. Nonetheless, mutant ESCs generated smaller teratomas with lower cell proliferation and higher propensity for cell death in vivo, reinforcing the notion that IMPDH cytoophidium is an important metabolic regulator, especially when nutrient supply is limited.

Mouse PSCs prefer glycolysis over oxidative phosphorylation for anabolic pathways to support rapid proliferation [24]. During somatic reprogramming, cytoophidium assembly initiates prior to the elevation of pluripotency gene expression, aligning with the upregulation of genes associated with metabolic reprogramming. In addition, we found that inhibiting glycolysis or PPP in ESCs leads to the breakdown of IMPDH cytoophidium. These results suggest that the formation of IMPDH cytoophidium could be triggered by an increase in metabolic flux towards purine nucleotide synthesis. This is in line with our previous study showing that the elevation of IMP, which is the substrate of IMPDH, promotes the assembly of cytoophidium in cells [11].

Our findings also show that the loss of IMPDH cytoophidium results in a reduction in the expression of genes associated with the glycolytic pathway, PPP, as well as *c-Myc*, which plays a key role in the regulation of purine biosynthesis pathways. The oncogene *c-Myc* is a well-known master regulator of cellular metabolism and proliferation that controls the transcription of a wide range of genes [39]. The decrease in *c-Myc* expression could directly contribute to the downregulation of glycolytic and PPP genes [40].

Interestingly, the overall reduction in the expression of these metabolic genes was restored by guanosine supplementation, implying a positive feedback loop from the intracellular GTP level to the regulation of *c-Myc.* Hence, we propose that IMPDH polymerisation is crucial for facilitating the activation of the feedback loop between purine nucleotide synthesis and its upstream pathways.

Recently, the significance of elevated IMPDH levels in proliferative cells has been depicted in several studies. In various human cancers, the interplay between c-Myc and IMPDH has been demonstrated to regulate GTP metabolic reprogramming during tumorigenesis [41]. Elevated GTP production in human glioblastoma is essential for supporting rRNA and tRNA synthesis [42]. Similarly, the rRNA synthesis in activated T cells relies on high GTP production [43]. These observations emphasise the importance of maintaining the GTP pool in rapidly proliferating cells.

In this study, we show the occurrence of IMPDH cytoophidium assembly in implanted mouse embryos, and its increase during fetal development, proposing a pivotal role for IMPDH cytoophidium in fast-growing fetal cells. In fact, following implantation, active metabolism supplies energy production, anabolism and epigenetic regulation to support the rapid growth of tissues [44–46]. Isotope tracing and metabolomics studies have revealed that glucose-derived PPP fuels the expansion of purine nucleotides during post-implantation stages [38]. Interestingly, the detection of IMPDH cytoophidia aligns with the progression of fetal development, implying extensive IMPDH polymerisation in developing tissues.

It has been shown that the loss of pluripotency in PSCs diminishes their capacity to generate chimeric mice but does not reduce the birth rate of live pups [47]. Surprisingly, our IMPDH^Y12C^ ESCs not only failed to generate chimeric mice but also dramatically reduced the number of live births after blastocoel injection. Therefore, we propose that the pluripotency of IMPDH^Y12C^ ESCs may not be directly impacted and could contribute to the development of host embryos. However, defects in maintaining the GTP pool could impair crucial regulatory processes in fetal tissues, ultimately leading to the termination of development.

**Fig. 9:**
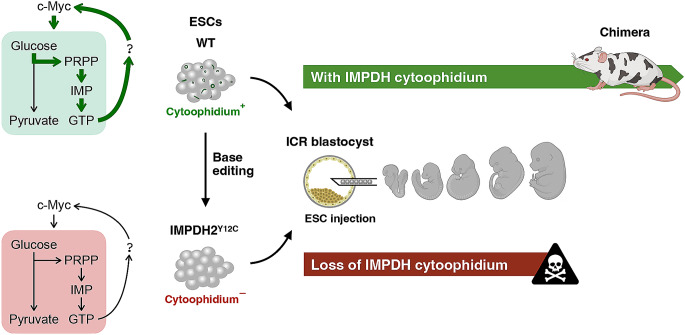
Schematic summary illustrating the involvement of IMPDH cytoophidium in the glycolysis-derived purine nucleotide synthesis pathway and mouse fetal development

Taken together, our study illustrates a positive correlation between IMPDH cytoophidium assembly and active glycolytic metabolism (Fig. 9). The absence of IMPDH polymers in IMPDH^Y12C^ ESCs results in downregulated *c-Myc* expression, potentially diminishing glycolysis and the metabolic flux towards nucleotide synthesis via the PPP. These findings shed light on the significance of IMPDH polymerisation in metabolic regulation and animal development. Future investigations are warranted to explore whether IMPDH polymerisation and cytoophidium formation also play a role in modulating metabolic gene expression in other physiological contexts, such as T cell activation and thymocyte development.

### Electronic supplementary material

Below is the link to the electronic supplementary material.

Supplementary Material 1
