# Supplementary figures and images for: The IMPDH cytoophidium couples metabolism and fetal development in mice
Source: Cell Mol Life Sci. 2024 May 8;81(1):210. doi: 10.1007/s00018-024-05233-z (PMC11078715; doi:10.1007/s00018-024-05233-z)

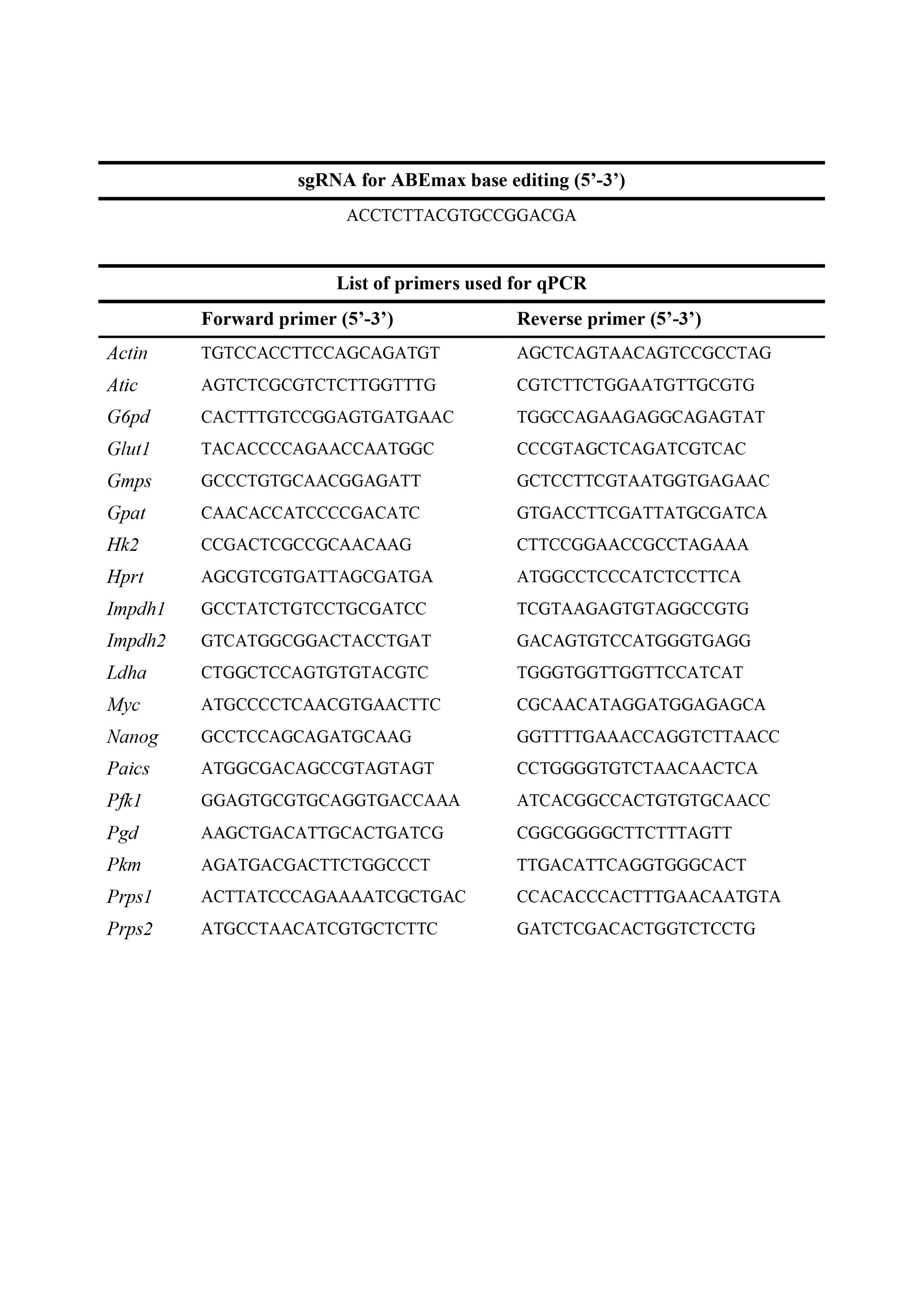

Supplement: Supplementary file 1 — Supplementary Material 1 [file 18_2024_5233_MOESM1_ESM.png]
